# Supplementary figures and images for: Molecular insights into postharvest seed coat darkening in common beans: a look beyond the P gene
Source: Front Plant Sci. 2025 Aug 19;16:1595906. doi: 10.3389/fpls.2025.1595906 (PMC12401897; doi:10.3389/fpls.2025.1595906)

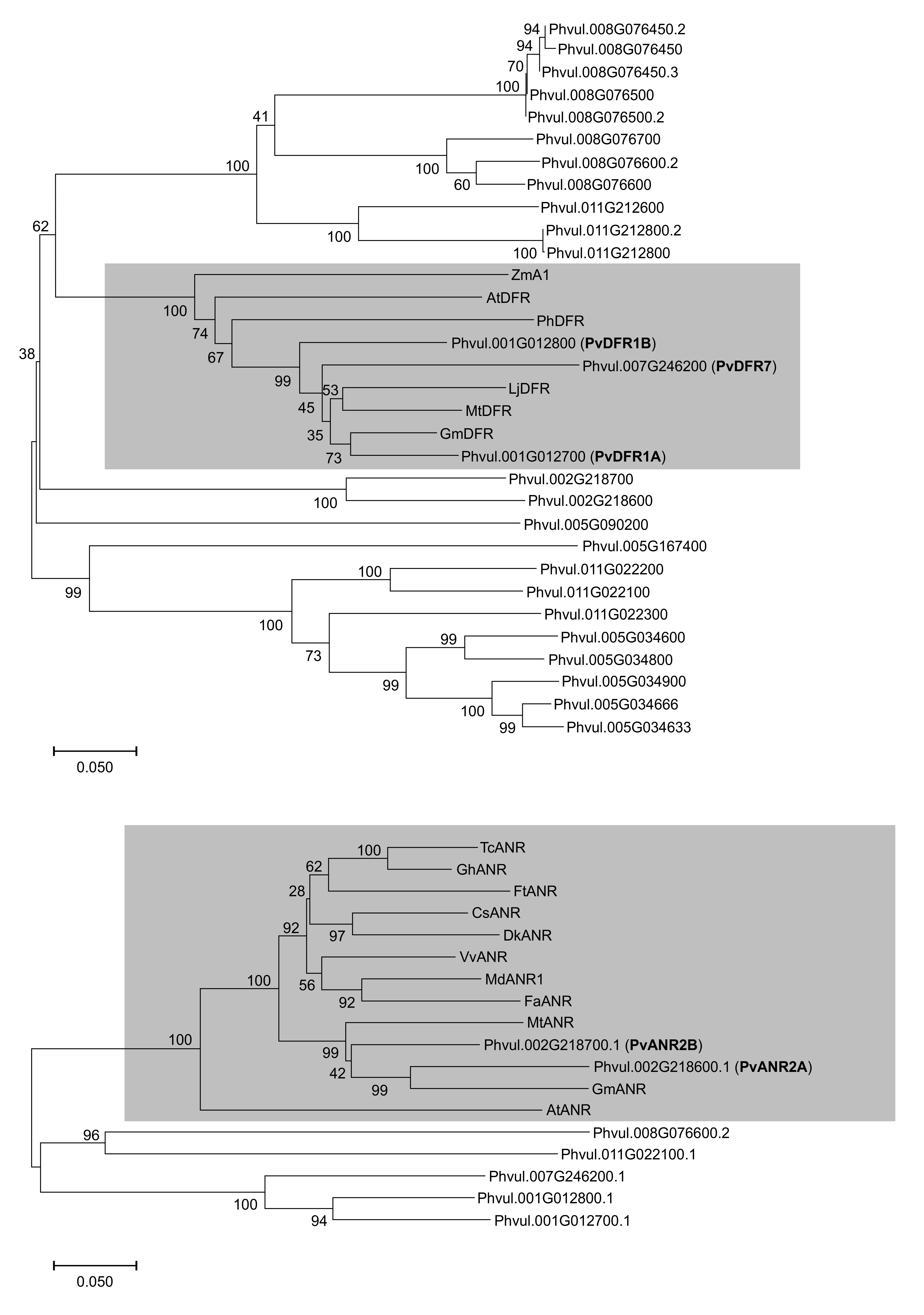

Supplement: Supplementary Figure 1 — Neighbor-joining trees of characterized DFR and ANR in legumes with common bean candidates. Amino acid sequences of PvDFR and PvANR from P. vulgaris (landrace G199833) were aligned with characterized DFRs and ANRs from other plants. Phylogenetic trees were built in MEGAX with bootstrap values (%; 1000 replicates) shown next to the branch points. Putative PvDFR and PvANR are shown in grey. (a) DFR tree: Compared proteins from Arabidopsis AtDFR (AT5G42800), G. max GmDFR (ABM64803), M. truncatula MtDFR (AY389346), Z. mays ZmA1 (CAA28734), Petunia x hybrida PhDFR (MW929212), L. japonicus LjDFR (BAE19950) with PvDFRs. (b) ANR tree: Compared proteins Arabidopsis AtANR (AT1G61720), G. max GmANR (Glyma08g06630), and M. truncatula MtANR (AAN77735), Fragaria x ananassa FaANR (ABG76842), Diospyros kaki DkANR (BAF56654), Gossypium hirsutum GhANR (ABM64802), Malus domestica MdANR1 (AAZ17408), Camellia sinensis CsANR (AAT68773), Theobroma cacao TcANR (ADD51354), Fagopyrum tataricum FtANR (AHA14497), Vitis vinifera VvANR (DQ129684) with PvANRs. [file Image1.tif]

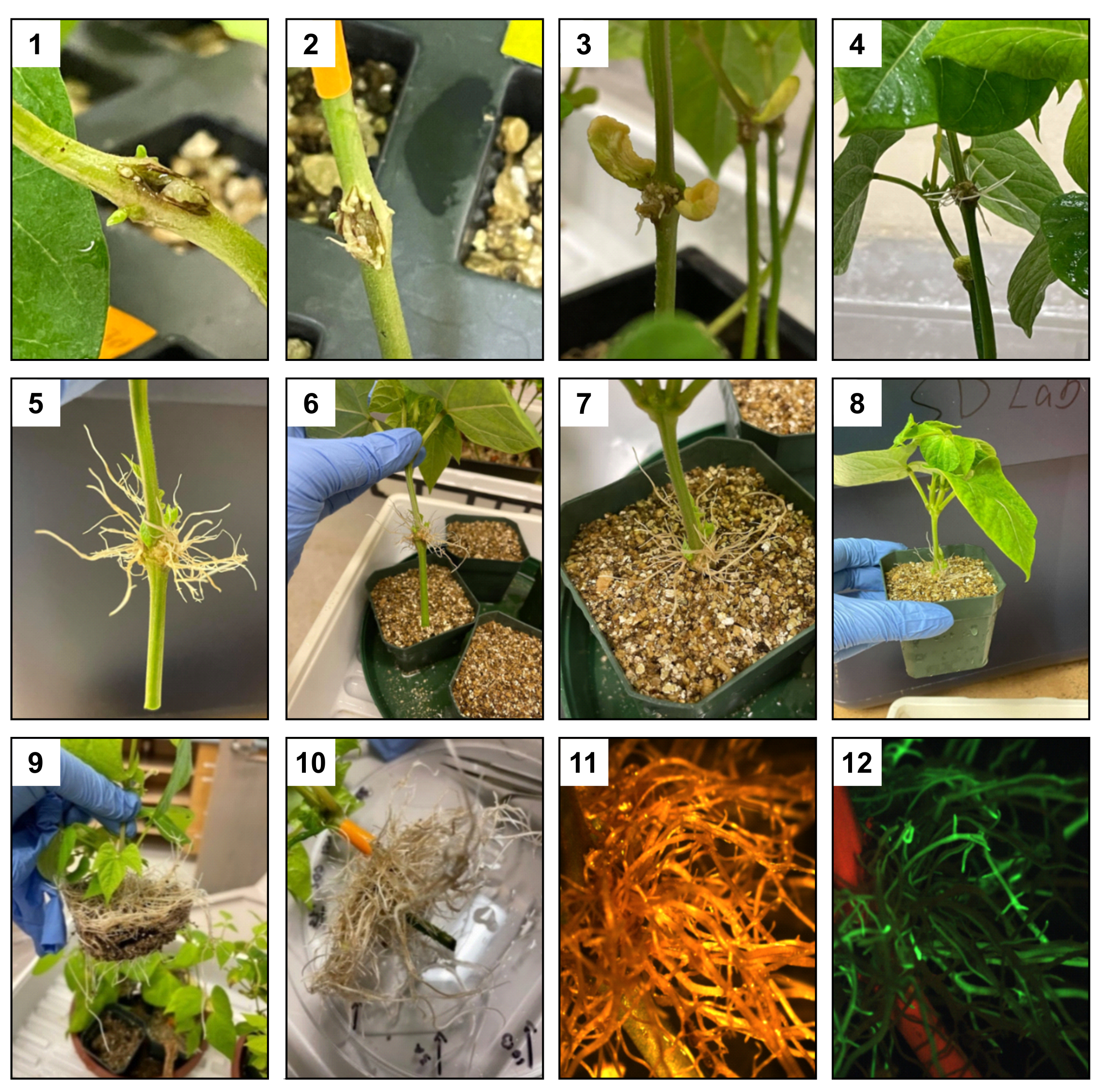

Supplement: Supplementary Figure 2 — Stages of hairy root generation in common bean. (1) callus formation at wounded sites at 5 days post-infection (dpi), (2) appearance of the first root from callus at 8–9 dpi, (3,4) roots growing at 11–14 dpi, (5-8) roots of length 1–2 cm ready to be transfered in a separate pot at 15 dpi by cutting 2 cm below the hairy roots, transplanted into a new pot, hairy roots were allowed grow over the vermiculite surface, (9) growth of hairy root at 18 dpi, (10) washed roots, (11) roots under a light microscope, (12) transgenic roots under UV-light showing GFP expression. [file Image2.tif]

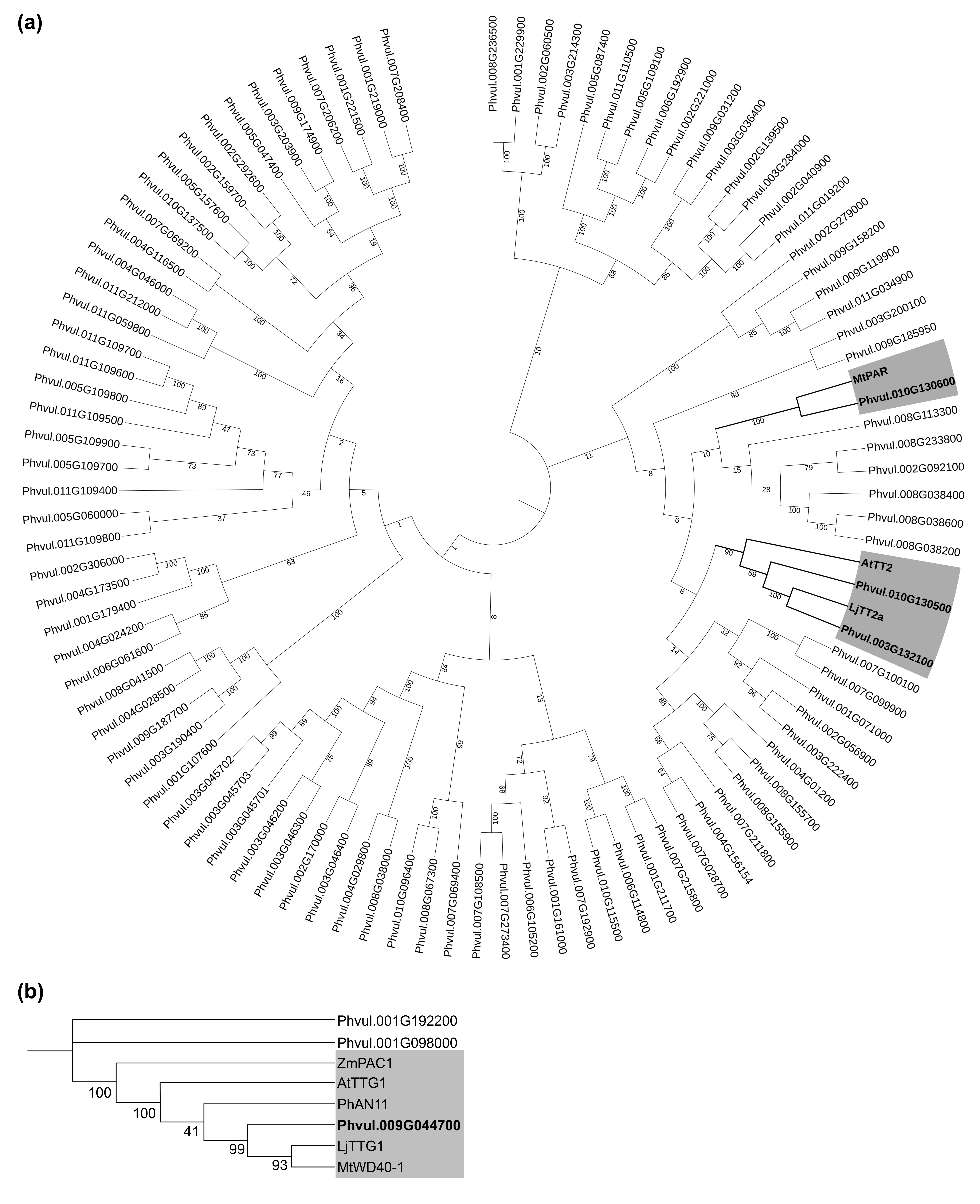

Supplement: Supplementary Figure 3 — Neighbor-joining trees of TT2 and TTG1 related MYB and WD40 proteins with common bean candidates. Trees were built using MEGAX software. Bootstrap values (%; 1000 replicates) are shown next to the branch points. Protein sequence alignments were performed using ClustalW. Clustered candidates are shown in grey. (a) MYB tree: PvMYBs from P. vulgaris (landrace G199833) were compared with characterized TT2 like MYBs from other plants, Arabidopsis AtTT2 (AT5G35550.1), M. truncatula MtPAR (HQ337434), and L. japonicus LjTT2a (AB300033). (b) WD40 tree: PvWD40s from P. vulgaris (landrace G199833) were compared with TTG1 like WD40 proteins from other plants, Arabidopsis AtTTG1 (AT5G24520.1) Z. mays ZmPAC1 (AY115485), P. hybrida PhAN11 (U94748), L. japonicas LjTTG1 (AB490777), M. truncatula MtWD40-1 (EU040206). [file Image3.tif]

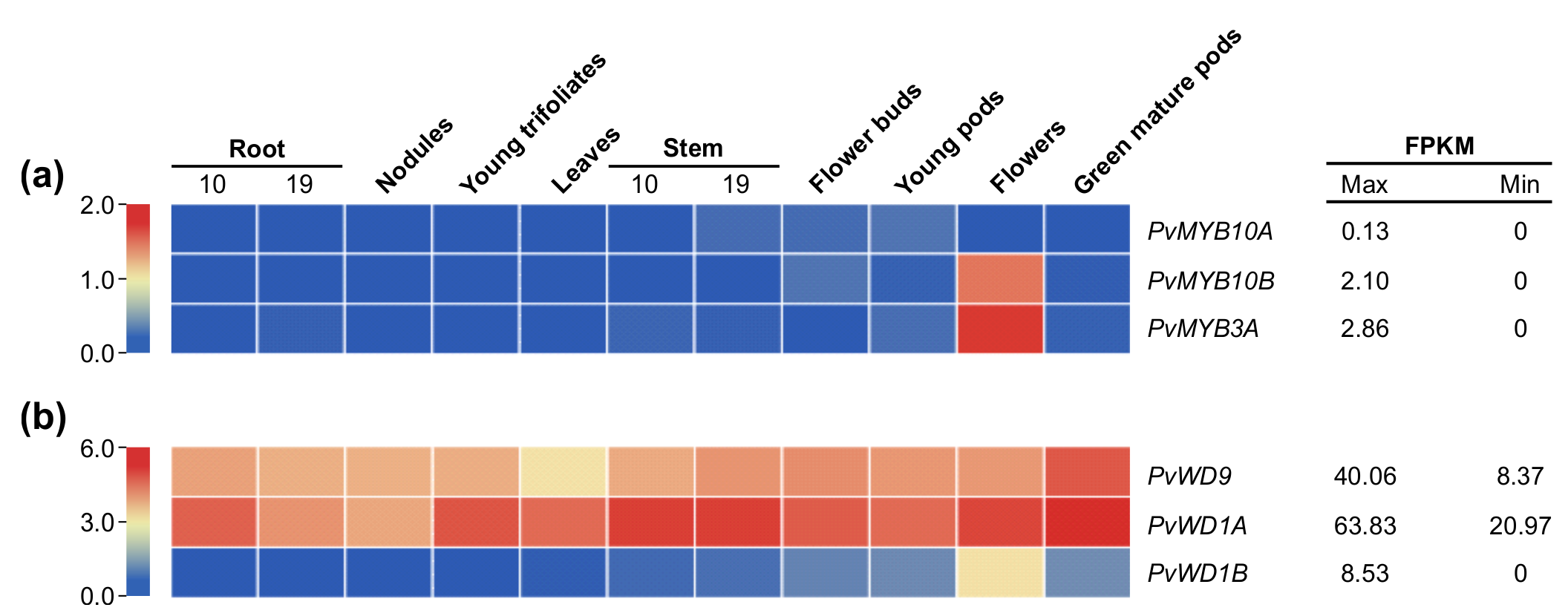

Supplement: Supplementary Figure 4 — Expression analysis of PvMYB and PvWD40 candidates in different tissues of common bean. Tissue-specific expression profile of the target genes in common bean. Transcript data retrieved from Phytozome for landrace G19833 was used for the generation of heatmap. The color key represents the relative transcript abundance in a row with the log2 transformed values. (a) MYB candidates, (b) WD40 candidates. [file Image4.tif]

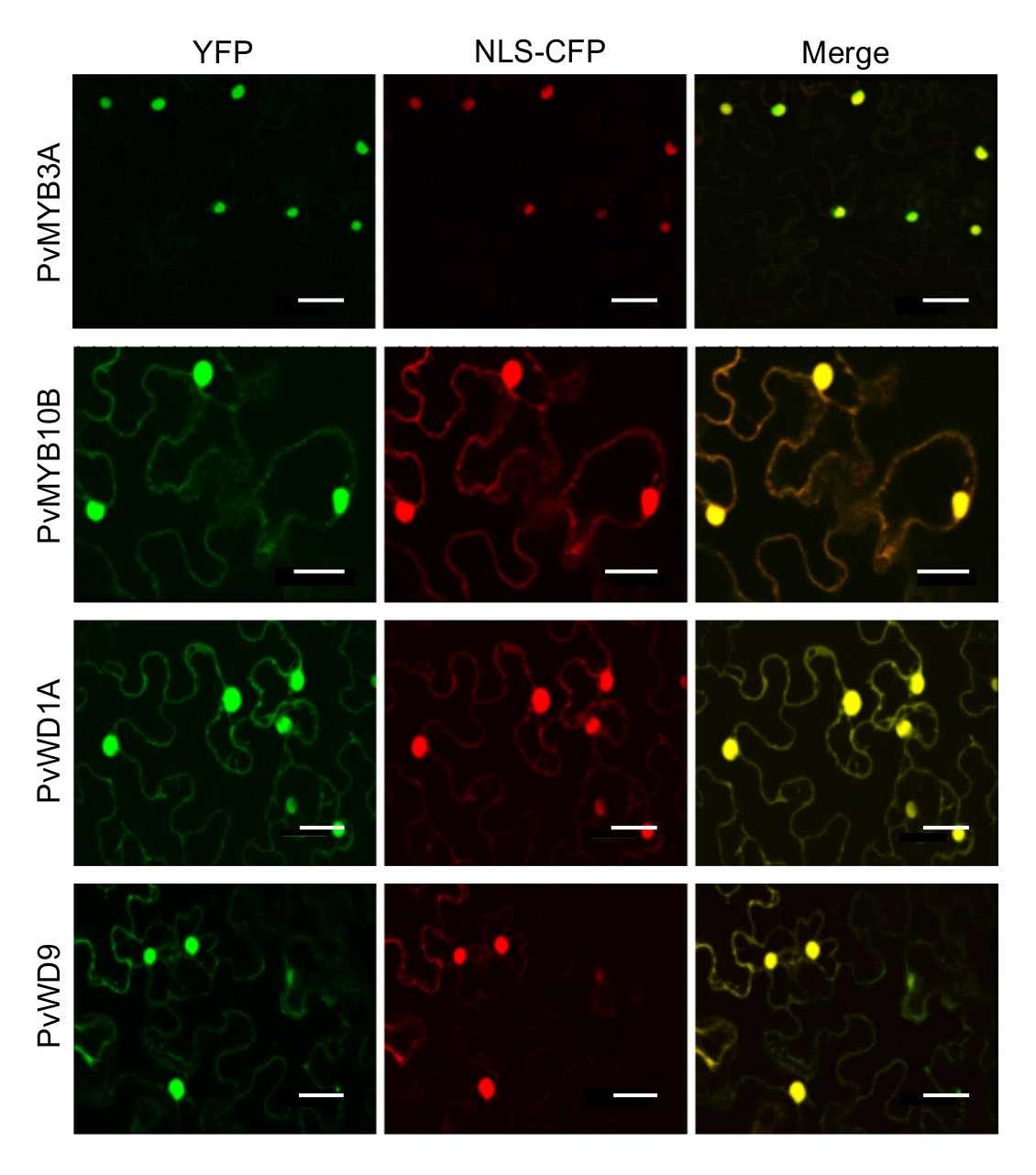

Supplement: Supplementary Figure 5 — Subcellular localization of the candidate interactors of P, PvMYB3A, PvMYB10B, PvWD1A and PvWD9 were fused upstream of YFP reporter gene, co-transformed with NLS-CFP into N. benthamiana. Fluorescence were visualized in leaf epithelial cells by confocal microscopy. Merged signal was collected by sequential scanning of YFP and CFP channels. Scale bar=30 μm. [file Image5.tif]

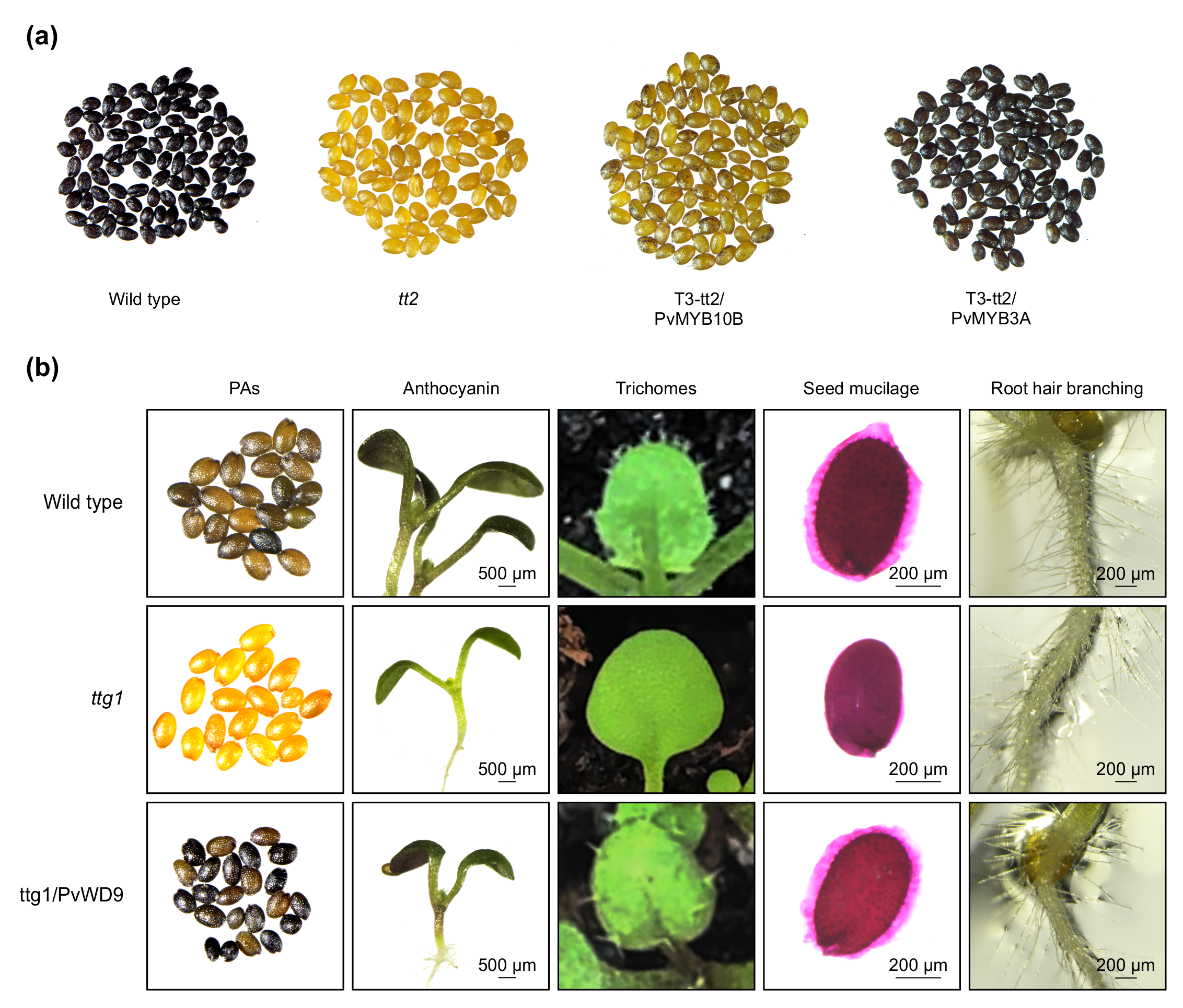

Supplement: Supplementary Figure 6 — Protein-protein interaction between candidate proteins involved in the MBW complex formation in common bean. BiFC assay between proteins was assayed by co-expression of translational fusions with N–terminal (YN) and C–terminal (YC) fragments of YFP in N. benthamiana. Proximity of the two fragments results in a functioning fluorophore. Scale bar= 40 µM. [file Image6.tif]

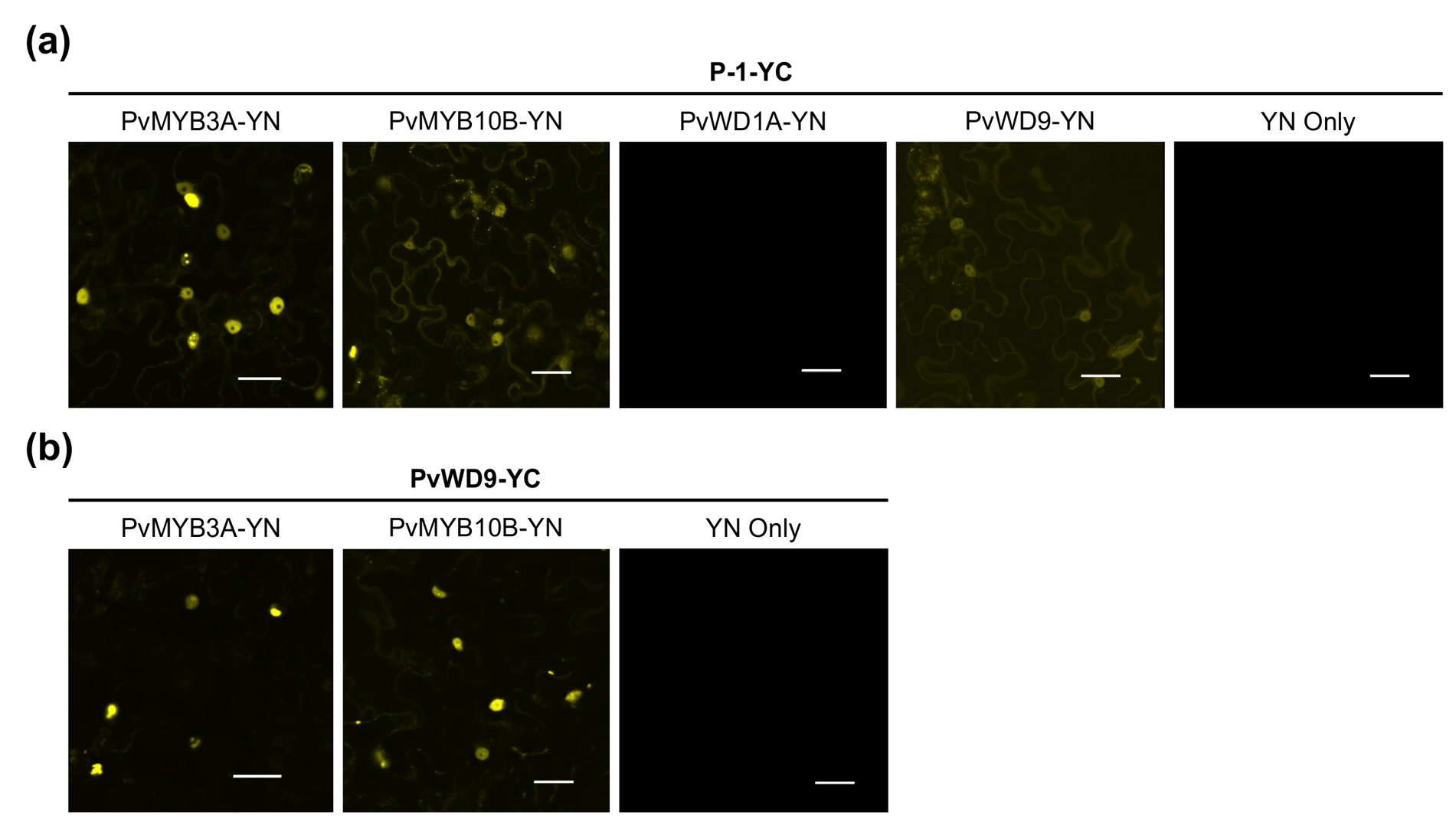

Supplement: Supplementary Figure 7 — Functional analysis of PvMYB3A, PvMYB10B and PvWD9 using Arabidopsis mutants. (a) DMACA stained seed of WS2, tt2, tt2 transformed lines with PvMYB10B, tt2 transformed lines with PvMYB3A, (b) Genetic complementation of ttg1 in Arabidopsis by variants of PvWD9. Phenotypes of Columbia-0, ttg1 and ttg1 transformed lines with PvWD9 are shown. From top, photographs of DMACA stained seeds, Anthocyanin in seedlings, trichrome in young leaf, Ruthenium red stained seeds showing seed mucilage and root hair patters. [file Image7.tif]
