# Supplementary material for: Molecular insights into postharvest seed coat darkening in common beans: a look beyond the P gene
Source: Front Plant Sci. 2025 Aug 19;16:1595906. doi: 10.3389/fpls.2025.1595906 (PMC12401897; doi:10.3389/fpls.2025.1595906)
Supplement: Supplementary Table 2 — Cis-acting elements in PvUANR2B promoter. [file Table2.docx]

**Table S2.** Position pf the c*is*-acting elements of *PvUANR2B* promoter.

| **Transcription Factor** | ***Cis* element** | **Sequence** | **Position** |
| --- | --- | --- | --- |
| bHLH | MYCCONSENSUSAT/ E-BOX | CANNTG | -1397 (+, -) |
|  |  |  | -761 (+, -) |
|  |  |  | -223 (+, -) |
|  |  |  | -147 (+, -) |
| MYB | MYB1AT | WAACCA | -1947 (+) |
|  | MYB2CONSENSUSAT | YAACKG | -1884 (-) |
|  | MYBCORE | CNGTTR | -1884 (+) |
|  | MYB2AT | TAACTG | -1884 (-) |
|  | MYB2CONSENSUSAT | YAACKG | -1774 (-) |
|  | MYBCORE | CNGTTR | -1774 (+) |
|  | MYB2AT | TAACTG | -1774 (-) |
|  | MYB2CONSENSUSAT | YAACKG | -1763 (-) |
|  | MYBCORE | CNGTTR | -1763 (+) |
|  | MYBCOREATCYCB1 | AACGG | -1763 (-) |
|  | MYB1AT | WAACCA | -1448 (-) |
|  | MYBST1 | GGATA | -1433 (+) |
|  | MYB1AT | WAACCA | -1429 (+) |
|  | MYBCORE | CNGTTR | -1151 (-) |
|  | MYB2AT | TAACTG | -1151 (+) |
|  | MYB2CONSENSUSAT | YAACKG | -1151 (+) |
|  | MYB1AT | WAACCA | -1120(+) |
|  | MYB1AT | WAACCA | -963 (-) |
|  | MYBATRD22 | CTAACCA | -962 (-) |
|  | MYB1LEPR | GTTAGTT | -180 (+) |
|  | MYB1AT | WAACCA | -164 (+) |
|  | MYBPLANT | MACCWAMC | -158 (+) |
|  | MYBCOREATCYCB1 | AACGG | -112 (+) |
